# Priapism secondary to antipsychotic treatments with favorable response to amisulpride

**DOI:** 10.1192/j.eurpsy.2022.1873

**Published:** 2022-09-01

**Authors:** A. Pérez Balaguer, B. Sanz-Aranguez-Ávila, B. Estévez-Peña, P. Fernández-Guisasola, E. Moyano-Ramírez

**Affiliations:** Hospital Universitario Puerta de Hierro de Majadahonda, Psychiatry, Majadahonda, Spain

**Keywords:** schizophrénia, Priapism, alpha-1-adrenergic, antipsychotic

## Abstract

**Introduction:**

Priapism is an abnormally prolonged erection, painful and irreducible, unrelated to sexual stimulation. Around 25-40% of cases are iatrogenic, especially associated with pharmacological treatments, of which antipsychotics (first and second generation) account for 50%.

**Objectives:**

The aim is to discuss a clinical case to provide further evidence.

**Methods:**

The patient was a 37-year-old man with a diagnosis of schizophrenia who was admitted for clinical decompensation. He had stopped antipsychotic treatment three months earlier due to side effects. He reported previous episodes of priapism associated with Risperidone, Aripiprazole, Olanzapine, and Paliperidone. At admission, he was administered Asenapine 20mg with development of priapism. Treatment was stopped. The urologists performed a lavage of the corpora cavernosa and administered adrenaline. In the absence of effectiveness, surgical intervention was successfully performed. Given the psychopathological improvement, he was discharged without antipsychotic treatment and close follow-up.

**Results:**

He presented a new admission one month later. Amisulpride was prescribed up to 800mg/day with good evolution and no adverse effects.

**Conclusions:**

Antipsychotic-induced priapism appears to be related to the blockade of alpha-1 adrenergic receptors in the corpora cavernosa. There is a positive correlation between the affinity for the receptor and the propensity to cause priapism. The dose and duration of the medication do not appear to be correlated. Other risk factors are a history of previous episodes, restarting medication after noncompliance, use of concomitant substances or medications that cause priapism. Our choice of amisulpride was based on the fact that it has no affinity for alpha-1 adrenergic receptors.

**Disclosure:**

No significant relationships.

